# Human Papilloma Virus self-sampling performance in low- and middle-income countries

**DOI:** 10.1186/s12905-020-01158-4

**Published:** 2021-01-06

**Authors:** Ashwini Kamath Mulki, Mellissa Withers

**Affiliations:** 1 Department of Family Medicine, Lehigh Valley Health Network, 1730 Chew St, Allentown, PA 18104 USA; 2 Keck School of Medicine, University of Southern California, 2001 N Soto Street SSB 318G, Los Angeles, CA 90032 USA

**Keywords:** HPV, Screening, Self-sampling, Cervical cancer

## Abstract

**Background:**

Screening for HPV has led to significant reductions in cervical cancer deaths in high-income countries. However, the same results have not been achieved in low- and middle-income countries (LMICs). HPV self-sampling is a novel approach that could improve screening rates.

**Methods:**

This study’s objective is to summarize the recent literature on HPV self-sampling in LMICs, focusing on sensitivity/specificity, and feasibility/acceptability of self-sampling compared to traditional screening methods. We conducted a PubMed search for articles published in English within the last 10 years on self-sampling in LMICs.

**Results:**

Fifty eligible articles from 26 countries were included, 19 of which came from sub-Saharan Africa and 18 from Latin America/Caribbean. Seven studies examined sensitivity, with five reporting rates higher than 91%. Six reported on specificity, which was also very high at 86–97.8%. Six studies examined self-sampling concordance with provider-collected sampling, with concordance rates ranging from 87 to 97.5%. A total of 38 studies examined the feasibility/acceptability of HPV self-sampling. Participation rates were very high in all studies, even when self-sampling was done at participants’ homes (over 89% participation). Overall, participants reported that HPV self-sampling was easy to perform (75–97%, 18 studies), painless (60–90%, nine studies), and preferred over provider-collected sampling (57–100%, 14 studies). Eight studies reported follow-up rates for participants who completed self-sampling; however, these rates varied widely-from 13.7 to 90%. The major benefits of self-sampling include convenience of screening from home, less embarrassment, and less travel. Improved education and awareness of self-sampling, combined with support from community health workers, could reduce perceptions of self-sampling being inferior to provider-collected sampling. Improving follow-up of abnormal results and improving linkages to treatment are also essential.

**Conclusion:**

Our literature review highlights HPV self-sampling is a well-performing test that shows promise in terms of expanding screening efforts for the prevention of cervical cancer-related deaths in LMICs.

## Background

Cervical cancer is the fourth most common cancer in women globally; in 2018 approximately 311,000 deaths around the world were attributed to cervical cancer, with over 90% of these occurring in low- and middle-income countries (LMICs) [1]. Human Papilloma Virus (HPV), a common sexually transmitted virus, is responsible for over 90% of cervical cancer [2].

Cervical cancer is a preventable disease. Pap smear, visual inspection with acetic acid (VIA) and visual inspection with Lugol’s iodine (VILI) have been the standard screening methods for decades in many countries. Screening has led to significant reductions in cervical cancer deaths in high-income countries. However, its impact on reducing cervical cancer related mortality in LMICs has been low due to several reasons [3]. The cost of screening equipment, the need for trained staff to provide traditional services, and low availability of screening have been identified as health systems-related reasons for low screening rates [4]. Poor access to health care, high stigma, low awareness on benefits of early screening, wait times, embarrassment and violation of privacy, and need for spousal permission contribute to avoidance of cervical cancer screening by women [5].

Human Papilloma Virus (HPV) self-sampling has been advocated as a novel way of addressing these concerns. Since 2013, the World Health Organization (WHO) has recommended HPV self-sampling as an option for initial screening; those who screen positive on this initial screening will then undergo more extensive testing. HPV self-sampling is a promising strategy to overcome the multiple barriers to cervical cancer screening in low-resource settings. However, it is unclear if this method will be suitable for LMIC settings. The purpose of this study is to summarize the results of all available studies on HPV self-sampling in the last decade (Jan 2010 to Dec 2019) in LMICs. In particular, we look into the sensitivity, specificity, feasibility and acceptability of HPV self-sampling compared to traditional cervical cancer screening methods.

## Methods

**Fig. 1:**
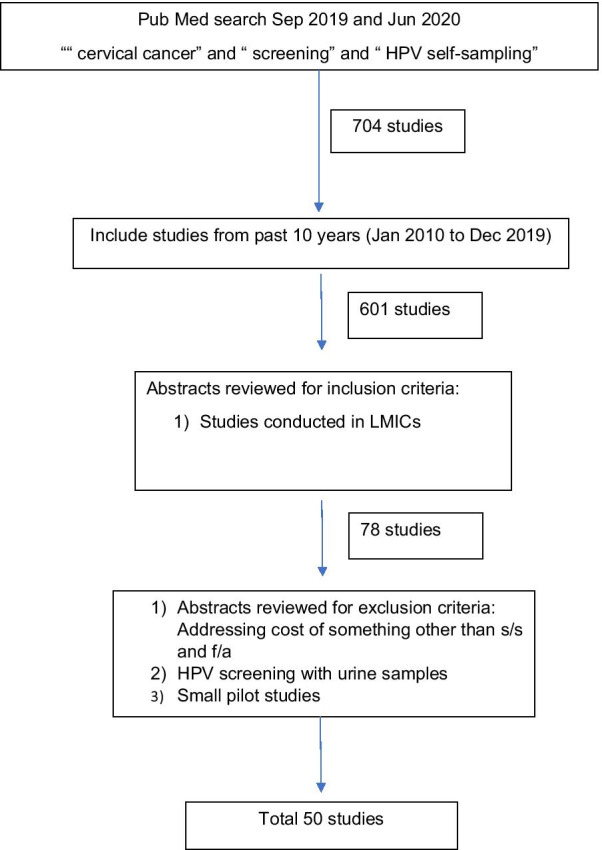
Selection process

As seen in Fig. 1, we conducted a search in PubMed using the words, “cervical cancer” and “screening” and “HPV self-sampling”, which yielded 704 studies. Two reviewers completed this search in September 2019 and then again in June 2020 to ensure all relevant articles were included. Among these, 601 articles were from past 10 years. Abstracts were reviewed to further narrow studies to those conducted in LMICs (per the World Bank Income classification, 2019) and in English, which yielded 78 articles [6]. We excluded studies addressing cost, urine samples, small pilot studies, articles in language other than English, and those with unclear methodology. Fifty articles remained that met our criteria [7–56]. We grouped these studies into two categories: “sensitivity and specificity of HPV self-sampling”, and “feasibility and acceptability of HPV self-sampling”.

## Results

**Fig. 2:**
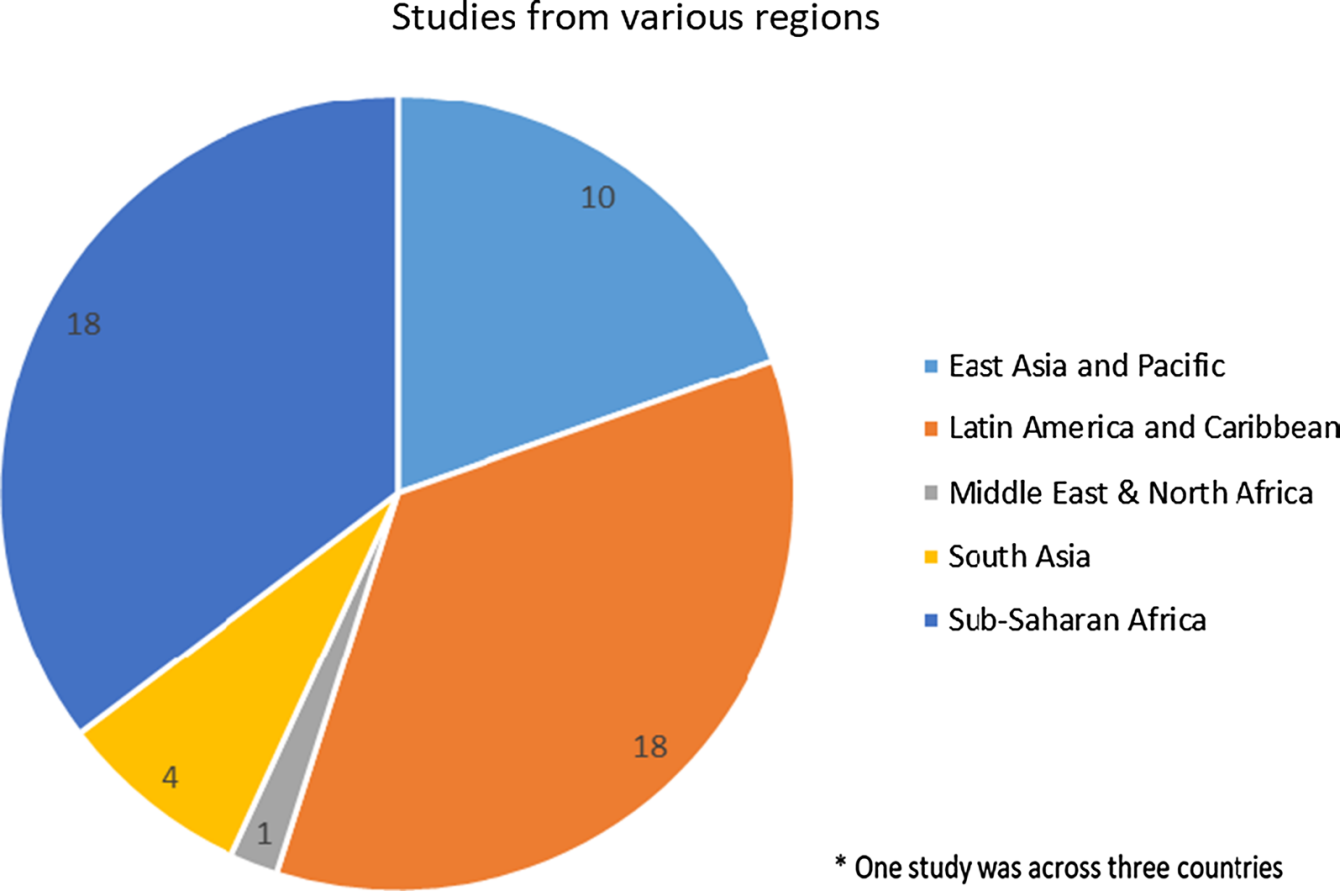
Study locations (regions per the World Bank Income classification, 2019)

**Fig. 3:**
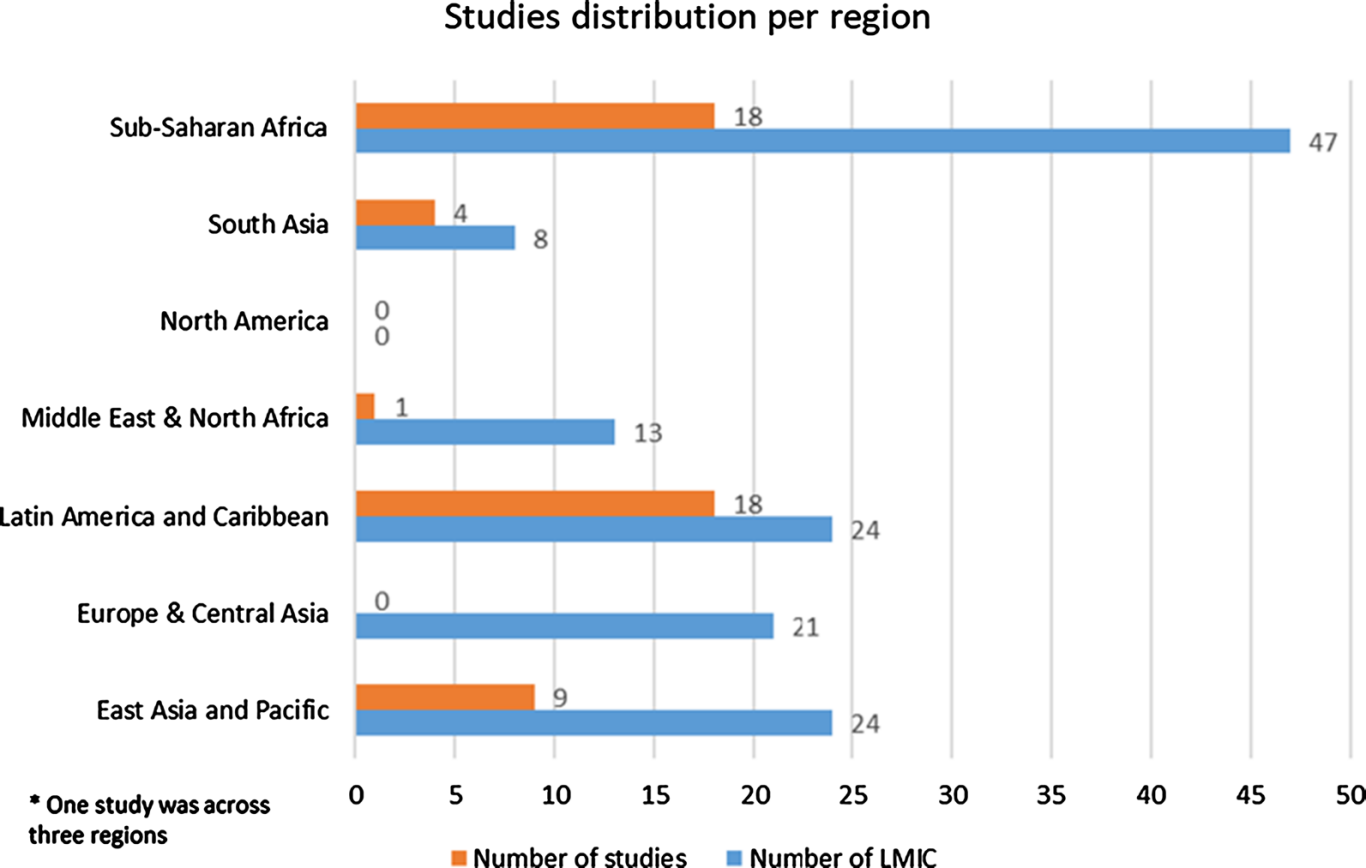
Distribution of studies per region (regions per the World Bank Income classification, 2019)

As seen in Fig. 2, this review included articles from 26 LMICs. Eighteen articles related to studies in sub-Saharan Africa, 18 in Latin America/Caribbean, 10 in East Asia/Pacific, 4 in South Asia, and one in Middle East/North Africa. As seen in Table 1 and Fig. 3, Latin America was widely represented, with 18 studies from only 8 of 24 eligible LMICs. South Asia and sub-Saharan Africa were also widely represented. Conversely, although there are 21 LMICs in the Europe/Central Asia region, zero studies came from this region. East Asia and Pacific had a poor representation with 10 studies from only 3 of the eligible 24 LMICs. And only one study came from the Middle East region, despite the fact that there are 13 LMICs in the region.

**Table 1:** Total LMICs and studies included in each region

Region	Number of LMIC	Number of studies
East Asia and Pacific	24	10
	China	3
	Thailand	5
	Malaysia	2
Europe & Central Asia	21	0
Latin America and Caribbean	24	18
	Haiti	2
	Mexico	4
	Peru	2
	Bolivia	1
	Brazil	3
	Guatemala	2
	El Salvador	2
	Nicaragua	2
Middle East & North Africa	13	1
	Egypt	1
North America	0	0
South Asia	8	4
	Nepal	1
	India	2
	Bhutan	1
Sub-Saharan Africa	47	18
	Malawi	1
	South Africa	2
	Ghana	1
	Kenya	4
	Cameroon	2
	Ethiopia	2
	Madagascar	1
	Senegal	1
	Zimbabwe	1
	Nigeria	1
	Uganda	2

**Fig. 4:**
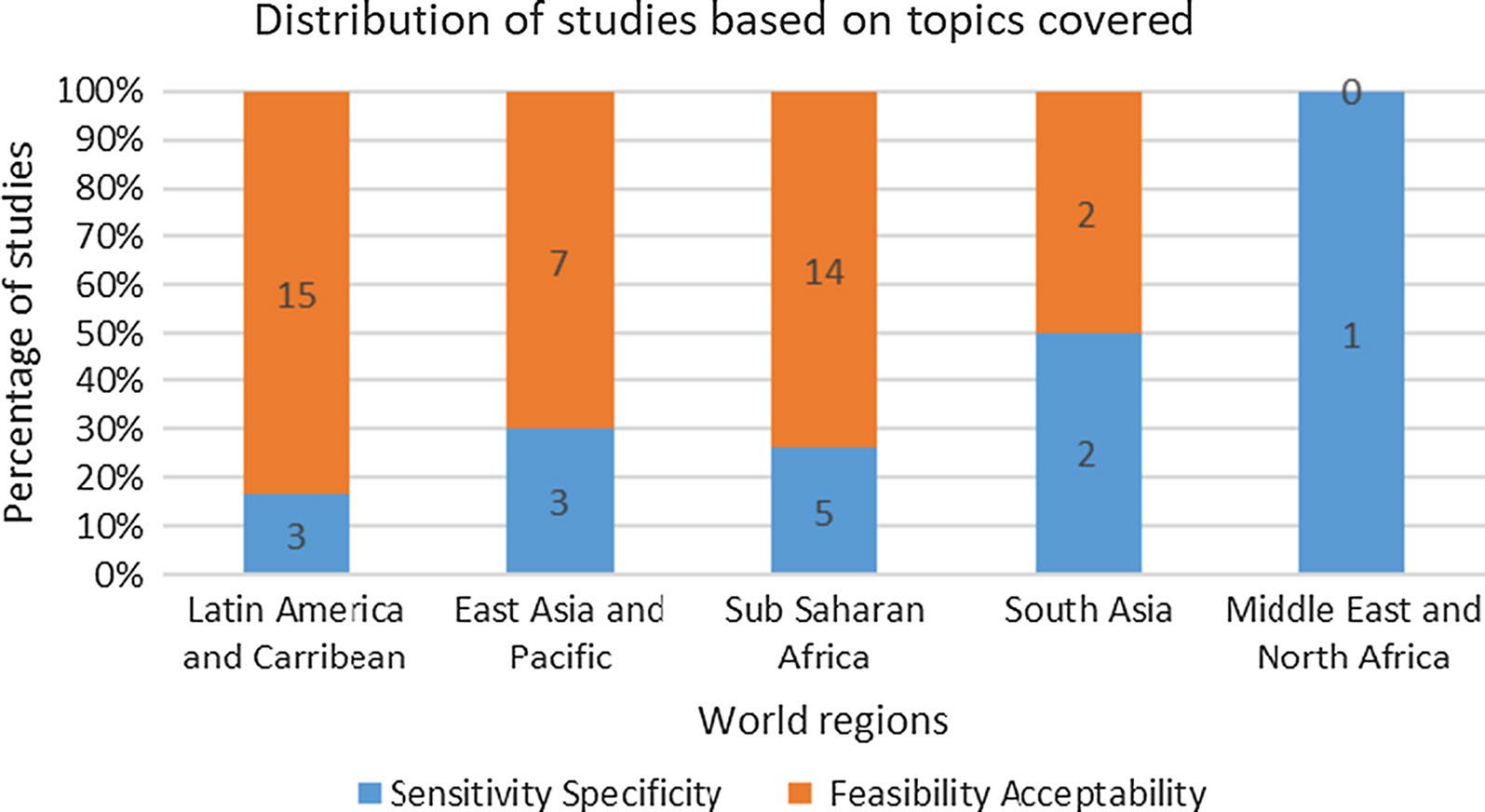
Distribution of studies based on topics covered. *2 studies, from India and Ghana, included both s/s and a/f

Fourteen studies reported on sensitivity and/or specificity and 38 studies looked at on feasibility and/or acceptability. Two studies included all of these topics, one in India and another in Ghana [7, 8]. Figure 4 shows the distribution of studies by region based on these two categories.

**Fig. 5:**
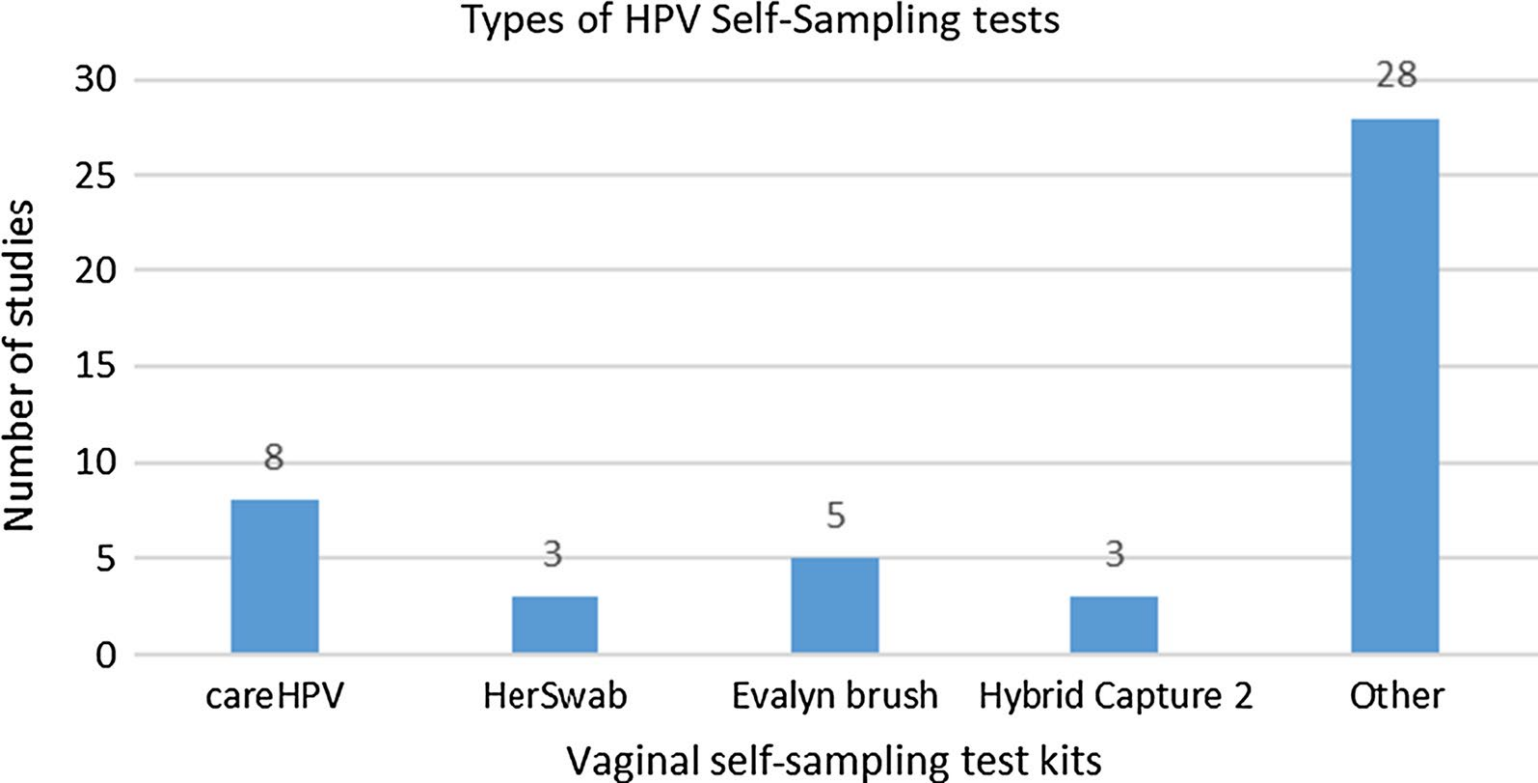
Types of HPV self-sampling tests

**Fig. 6:**
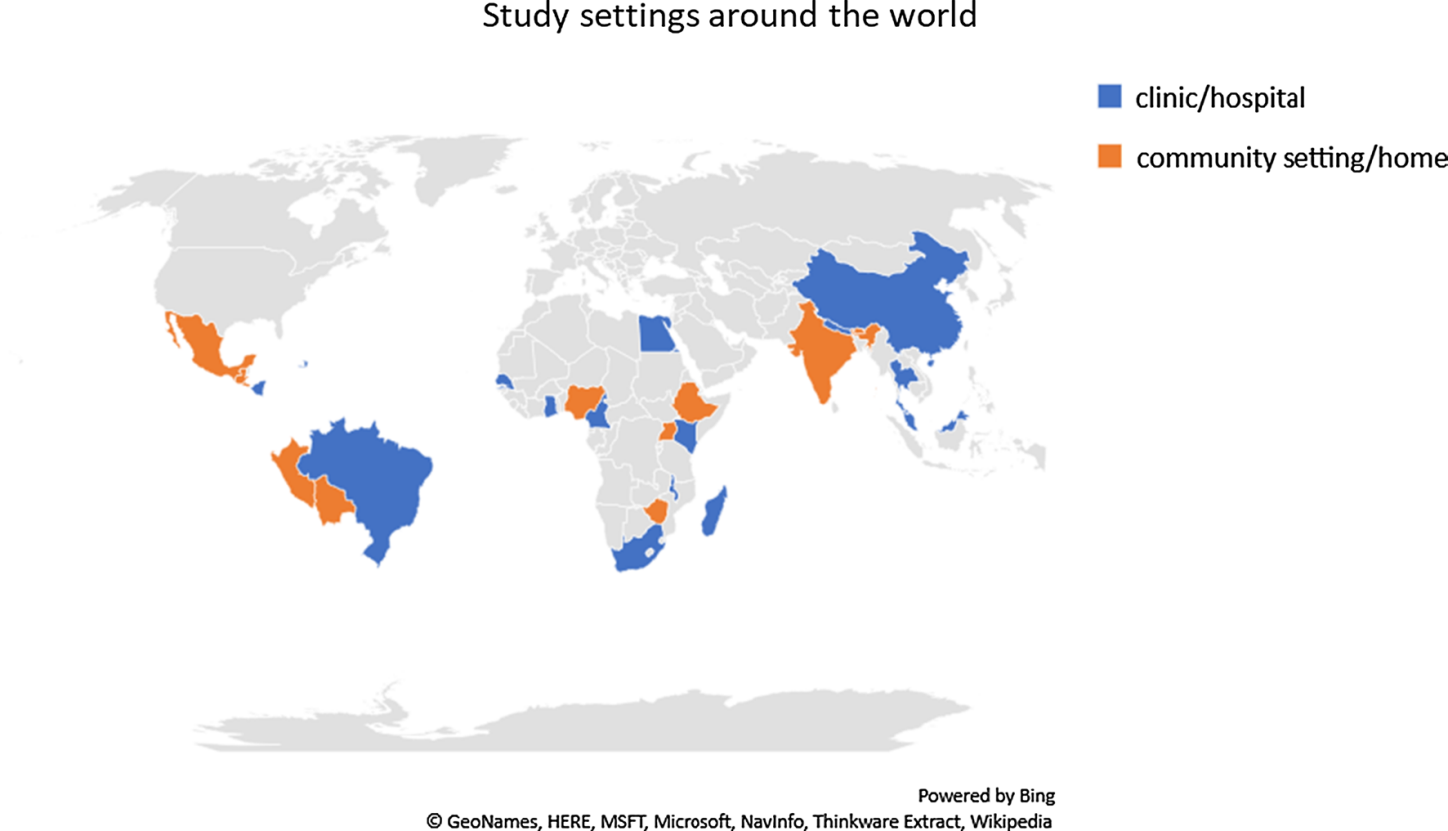
Study settings around the world

All of the studies except two included women performing HPV self-sampling. In the two studies, women did not actually perform self-sampling, but they were interviewed about their perceived benefits and barriers to this method [9, 10]. Device used for self-sampling was a (cervico-) vaginal swab or brush in all our studies. None of them included spatula or cervico-vaginal lavage. There were a variety of tests kits used and Fig. 5 highlights the common tests used. As seen in Fig. 6, HPV self-sampling was done at health centers (local clinics and hospitals) in 30 of the studies, while 20 studies were conducted at participants’ homes or in community settings such as schools or workplaces.

### Sensitivity and specificity

**Fig. 7:**
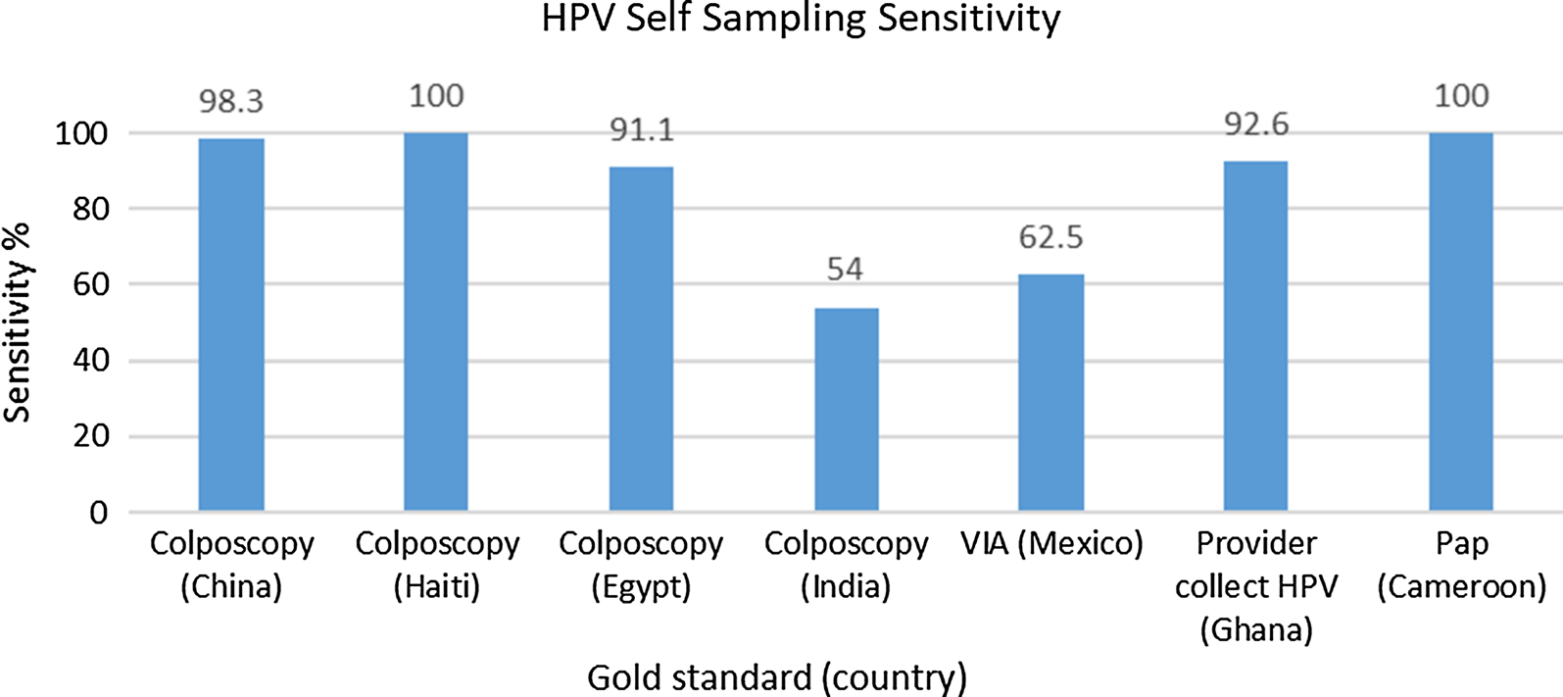
HPV self-sampling Sensitivity (*CIN 3 or greater for colposcopy and VIA, HGSIL for pap). *CIN, Cervical Intraepithelial Neoplasia; HGSIL, High Grade Squamous Intraepithelial Lesion

**Fig. 8:**
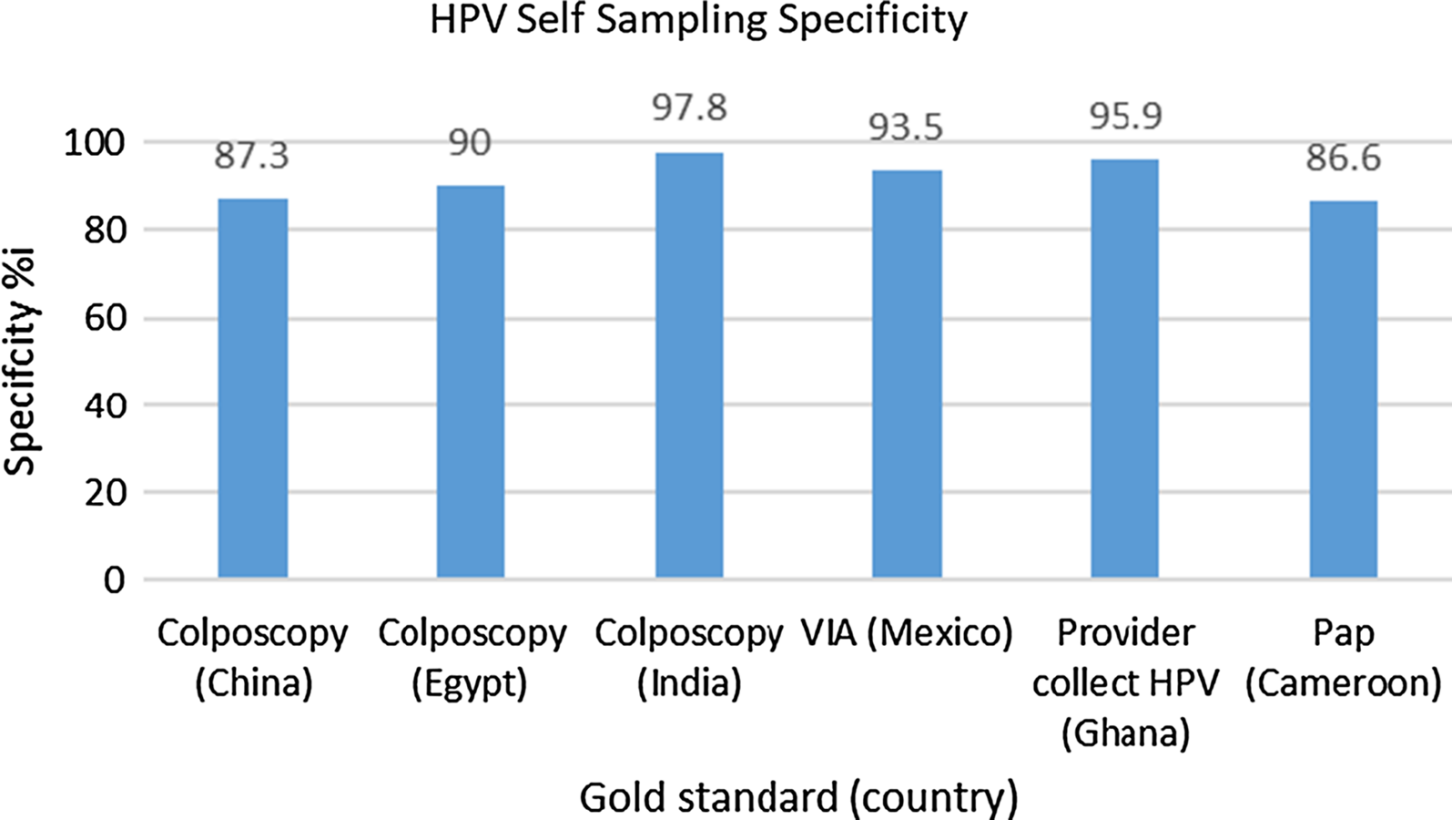
HPV self-sampling Specificity (*CIN 3 or greater for colposcopy and VIA, HGSIL for pap)

Among the 14 studies on sensitivity, specificity, and/or concordance with provider-collected samples, ten were cross-sectional studies, one was a randomized controlled trial, one was a prospective cohort study, and two were case control studies. Participant ages were between 25 and 65 years in 43% of studies, between 16 and 75 years in 43% of studies, while one study only recruited 16–17 year olds, and another included women between the ages of 20–89 years. Overall, as seen in Figs. 7 and 8, overall, these studies demonstrated high levels of sensitivity and specificity of self-sampling.

Seven of the 14 studies reported on sensitivity, with five of these studies reporting rates greater than 91%. Colposcopy was used as a gold standard in four studies, while VIA, provider-collected HPV and pap smears were used in other three studies. One of the largest studies was a multisite population-based study by Belison et al. (2012) in China with 10,000 participants. They reported a sensitivity as high as 98.3% for CIN3 [11]. In this study, colposcopy with four biopsies and endocervical curettage was used as the gold standard and sensitivities for self-sampling were comparable to provider-collected sampling. Lazcano-Ponce et al.’s [12] conducted a community-based, randomized controlled trial in Mexico with 25,601 participants, which demonstrated that self-sampling had 2.4 times more relative sensitivity for CIN3 as compared to cytology [12]. In this study, among those recruited for self-sampling at home 98% participated, while among those recruited for pap at the local clinic only 87% participated.

In contrast to the above studies, Labani et al.’s [7] study of over 5000 participants in India reported a low sensitivity of 54% for self-sampling. The authors posited that a low incidence of CIN2 or greater in this population was the explanation for these low rates [7].

Six of the 14 studies reported on specificity, with very high rates–ranging from 86.6 to 97.8%. For example, Kamal et al.’s [13] study of 1601 participants in Egypt reported a specificity of 90% which improved to 99.4% [95% CI 93.5–96.1%, *p* < 0.001] when combined with VIA reducing colposcopy referral rate from 5.3% (HPV alone) to 2.5% [13]. The study in Cameroon by Untiet et al. [14] reported specificity of 86.6%,however, these results have to be considered with caution as they used pap smear, which is substandard to colposcopy, as the gold standard [14].

**Fig. 9:**
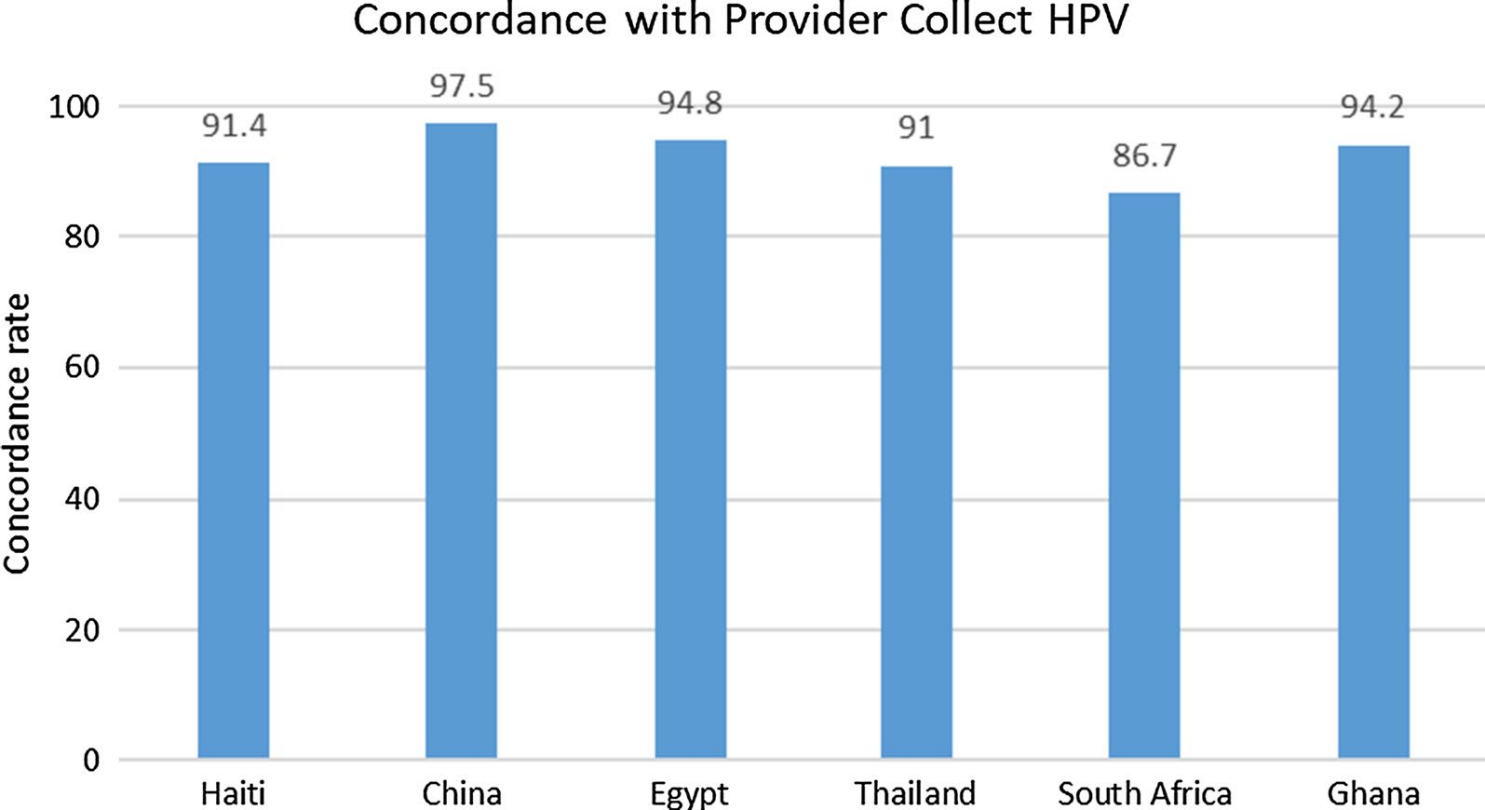
HPV self-sampling concordance with provider-collected sampling

As seen in Fig. 9, six studies reported HPV self-sampling concordance with provider-collected sampling. All reported very high concordance, ranging from 86.7 to 97.5%. Boggen et al.’s (2015) study of 1845 participants from Haiti had high concordance of 91.4% (κ = 0.73 (95% CI 0.69–0.77, *p* < 0.001)], similar to the study in Ghana by Obiri-Yeboah et al. [8] which reported a concordance rate of 94.2% [k = 0.88 (95%CI: 89.9–97.1, *p* < 0.0001)] [8, 15]. A slightly lower rate of 86.7% was found by Adler et al. [16] in South Africa where participants were adolescents in comparison to the adult participants in the other studies [16].

### Feasibility and acceptability

A total of 38 studies examined the feasibility and/or acceptability of HPV self-sampling. Five of these studies looked at acceptability, six studies were about feasibility, and 27 studies addressed both. Thirty-two studies were cross-sectional in design (three of these were mixed methods), four were randomized trials, and two were qualitative studies (interviews and focus groups). Participant ages were between 25 and 65 years in 68% of studies and between 16 and 75 years in 32% of studies.

Participation rate in self-sampling was very high in all studies. Six studies achieved a rate of 100%, while 17 studies were at 90–99%, five studies at 80–89%, one study at 70–79%, two studies at 60–69%, and seven studies did not mention participation rates. Among these, 10 were conducted at participants’ home, all of which reported participation rates of over 89%. Studies in Brazil and Uganda found that participation rates were significantly higher for self-sampling as compared to the standard screening methods, 100% (versus 60% pap) and 99.2% (versus 45.2% VIA) respectively [17, 18]. Studies from Ghana and Kenya showed that community-based self-sampling also had higher participation rates at 60% (Ghana) and 95.1% (Kenya) compared to 37% and 46.6% for hospital-based self-sampling [19, 20]. Baussano et al.’s [21] study of 3648 participants from Bhutan found that participation decreased with increases in travelling time from home to health centers,it was 90% (95% CI 84–94%) for women living less than 30 min from the health center but 62% (95% CI 50–73%) among those greater than or equal to six hours away from their homes [21].

**Fig. 10:**
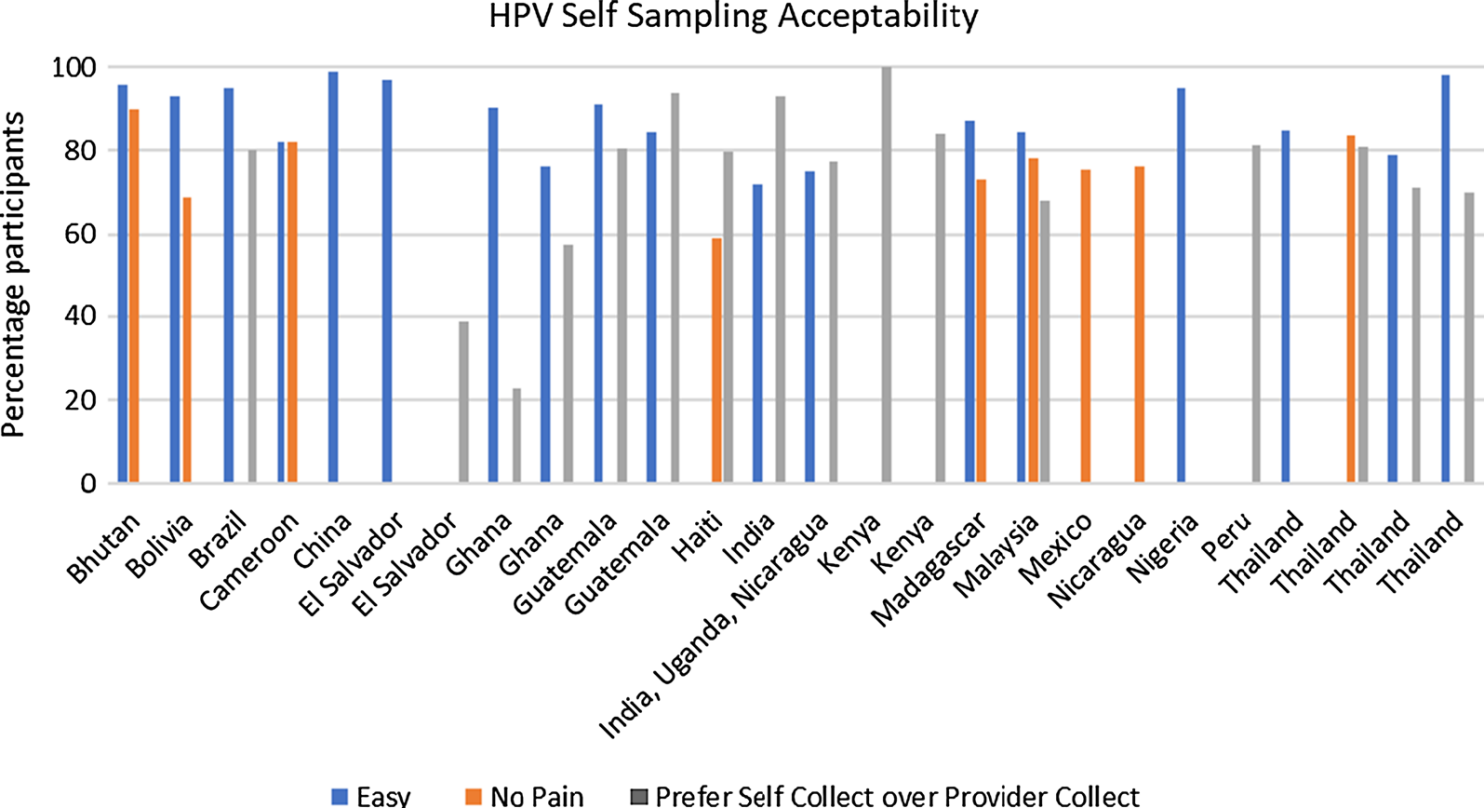
HPV self-sampling acceptability indicators

Overall, as seen in Fig. 10, the vast majority of studies reported positive feedback from participants regarding self-sampling; participants reported that HPV self-sampling was easy to perform (75–97%, 18 studies), painless (60–90%, nine studies) and preferred over provider-collected sampling (57–100%, 14 studies). In Arriba et al.’s [22] study of 2517 women in Mexico, 91% of women found self-sampling convenient, easy, and less embarrassing compared to other screening methods [22]. Similarly, Maza et al. (2017) in El Salvador found high satisfaction levels with self-sampling; in this study of 2019 women, 98.5% felt self-sampling saves time and 93.5% were less embarrassed doing the test themselves compared to provider sampling [23]. A mixed methods study by Bansil et al. [24] of 3863 participants from India, Nicaragua and Ghana found that 77.5% women preferred self-sampling over provider-collected sampling [24]. However, in this study over 50% of women reported fear of hurting themselves and the need for staff aid in self-sampling. This was similarly reported in the study by Arriba et al. [22], where 76.8% women preferred self-sampling at a clinic site over sampling at home because assistance would be available as needed at the clinic [22]. Participants from only two studies, in El Salvador and Ghana, reported low preference for self-sampling. Rosenbaum et al. [25] found that 29.3% of 518 women in El Salvador had no preference, 38.8% preferred self-sampling, and 31.9% preferred provider-collected sampling [25]. Reasons cited for preference of provider-collected sampling were perceived result accuracy (33%), provider’s knowledge (24.2%), practice or experience the provider had performing the procedure (16.4%), fear of improper sampling (13.3%), and comfort (7.9%). In rural Ghana, Awua et al. (2017) found that of the 415 participants, 22.6% preferred self-sampling compared to 56.2% who preferred provider-collected sampling, with two-thirds of this group believing that the provider collected a better sample [19].

Eight of the 38 studies reported follow-up rates for participants who completed self-sampling; follow-up rates ranged from 13.7 to 94.8%. Abuelo et al.’s [26] study in Peru reported that 81% of positive participants followed up for further testing [26]. Community health workers (CHWs) were actively engaged in this study from initial education on screening through follow-up. Gottschlich et al.’s [27] study of indigenous women in Guatemala also engaged CHWs, and demonstrated that 90% of participants called back for their results [27]. A study of 431 Thai women by Trope et al. [28] reported that 94.8% returned for results on same day. Ensuring that results were available within three hours of sample collection led to improved same-day follow-up in this study [28]. In contrast, Allende et al.’s [29] study in Bolivia, which included a self-sampling instruction card but no active CHW engagement in assistance and education of participants, found that only 13.7% positive participants returned for further testing [29]. Similarly in Kenya, where CHWs were not engaged, despite improved participation rates at community health campaigns compared to hospital services (60.0% vs 37.0%, *p* < 0.001), follow-up rates were low in both locations (CHCs 39.2%; health facilities 31.5%; *p* = 0.408), highlighting the need for improved linkage for follow-up and treatment [20].

## Discussion

The 2013 WHO guidelines on screening and treatment of precancerous lesions for cervical cancer prevention recommend HPV testing as the first screening method when feasible [4]. LMICs carry the greatest burden of cervical cancer and also report lower rates of screening as compared to high-income countries; our review from LMICs provides insight into the validity, acceptability and feasibility of self-sampling in these contexts.

Our results highlight that HPV self-sampling is a promising cervical cancer screening method in LMICs for several reasons. It is a valid test with high sensitivity and specificity. In fact, in over 70% of the studies reporting on validity in our review, self-sampling had a sensitivity and specificity of over 90%. The literature also demonstrated a very high concordance rate between self-sampling and provider-collected sampling, ranging from 86.7 to 97.5%. These results are consistent with the 2018 meta-analysis of 56 studies in under-screened women in developing countries. In this study HPV-self sampling was as accurate as clinical sampling with a pooled sensitivity of 99% (CI 0.97 to 1.02) and specificity of 85% (CI 0.80 to 0.89) [57]. However, it is important to note that validity may be affected by the type of brush or swab and the transportation medium used for collection and our review did not evaluate the details of these different testing materials.

The simplicity of self-sampling improves screening participation rates while also improving access to cervical cancer screening, making it particularly well-suited for low-resource settings. Participation rates in self-sampling were over 80% in over 90% of the studies, which increased to 89% when self-sampling was performed in participant homes. This aligns with the results of a 2019 meta-analysis, of 23 RCTs mostly from high-income countries, where women were twice as likely to participate in cervical cancer screening by HPV self-sampling compared to standard of care [58]. Our results showed that participants reported that the most important perceived benefits of self-sampling were the convenience of screening from home, less embarrassment, and less travel. However, some studies reported that women had concerns about the quality of self-sampling, privacy issues in sampling from home, and assistance with self-sampling as needed. Local schools and community health centers were utilized for mass community screenings in some studies. These may be acceptable alternatives for those concerned about cleanliness and privacy of self-sampling from home. Whenever possible, allowing for self-sampling to be done at home or at a community location with staff available to assist women is recommended.

Our results highlighted lack of follow-up as a major limitation of self-sampling. Follow-up rates must be improved in order to ensure that women who screen positive receive additional testing and treatment when necessary. Ensuring quick results of HPV self-sampling is key to expanding access to this option. Programs should provide single-visit, test-and-treat approaches to help reduce barriers to follow-up care, like transportation. Six studies found that engaging community health workers improved participation rates and significantly increased follow-up rates. CHWs are crucial in assisting with follow-up of abnormal results and improving linkage to further testing and treatment.

Our review also highlighted that the lack of affordable test equipment and financial support for training and employing CHWs to assist with screening and follow-up were potential barriers to the expansion of self-sampling cervical cancer screening programs in some studies. Improved education and awareness of sell-sampling methods, combined with support from CHWs, can help decrease participants’ perception of self-sampling being inferior to provider-collected sampling.

Several studies also underscored the role of culture in influencing women’s perceived benefits of screening, as well as the decision of who collects the sample and where it is collected. For example, some women required their husbands’ permission to participate in self-sampling programs while others mentioned a mistrust with local health systems as barriers to screening. Socio-cultural beliefs that may serve as barriers to self-sampling should be considered while designing cervical cancer screening programs.

Our results also underscored the need for additional research on self-sampling in LMICs. First, we found very few studies from LMICs evaluating validity. More studies are required across different LMICs to confirm self-sampling validity and to ensure reliability. In addition, our search found published studies on self-sampling from only 26 of the 137 LMICs in the past 10 years. Further, only four of the 10 countries with the highest rates of cervical cancer globally were represented, highlighting the dearth of research in this area [59]. More studies are needed to improve the applicability and generalizability of our results across different contexts.

## Conclusion

HPV self-sampling is a well-performing test that shows promise in terms of expanding screening efforts for the prevention of cervical cancer-related deaths in LMICs. Our study is a novel contribution because it synthesizes the evidence from LMICs, as well as examines four separate measures (S/S, F/A). We acknowledge the limitation of our study type in comparison to systematic reviews and meta-analysis which may help further strengthen the scientific value of our results. However, our results are similar to robust scientific studies conducted in higher income countries, which is promising. Our study can help inform public health and policy development to scale up efforts to reduce cervical cancer rates in LMICs.

Self-sampling is convenient, respects personal autonomy, reduces disparities to access to screening, and, most importantly, can help save women’s lives.

## Data Availability

All data generated or analyzed during this study are included in this published article.

## Abbreviations

LMIC: Low- middle-income country
HPV: Human Papilloma Virus
WHO: World Health Organization
VIA: Visual inspection with acetic acid
CIN: Cervical intraepithelial neoplasia
CHW: Community health worker
CHC: Community health campaign
